# Characterization of *Arcobacter* spp. Isolated from human diarrheal, non-diarrheal and food samples in Thailand

**DOI:** 10.1371/journal.pone.0246598

**Published:** 2021-02-05

**Authors:** Paksathorn Kietsiri, Chonchanok Muangnapoh, Woradee Lurchachaiwong, Paphavee Lertsethtakarn, Ladaporn Bodhidatta, Orasa Suthienkul, Norman C. Waters, Samandra T. Demons, Brian A. Vesely

**Affiliations:** 1 Department of Bacterial and Parasitic Diseases, Armed Forces Research Institute of Medical Sciences (AFRIMS), Bangkok, Thailand; 2 Department of Microbiology, Faculty of Public Health, Mahidol University, Bangkok, Thailand; Nitte University, INDIA

## Abstract

*Arcobacter butzleri* is an emerging zoonotic food-borne and water-borne pathogen that can cause diarrhea in humans. The global prevalence of *A*. *butzleri* infection is underestimated, and little is known about their phenotypic and genotypic characterization. The aim of this study was to determine antimicrobial susceptibility (AST) profiles, detect related virulence genes, and classify sequence type (ST) of *A*. *butzleri* isolates obtained from human stool and food samples. A total of 84 *A*. *butzleri* isolates were obtained from human diarrheal (n = 25), non-diarrheal (n = 24) stool, and food (n = 35) samples in Thailand. They were evaluated for phenotypic identification by conventional microbiological procedures and AST by Kirby-Bauer disc diffusion method as well as virulence genes detection. Representative isolates from each origin were selected based on the presence of virulence genes and AST profiles to analyze genetic diversity by multilocus sequence typing (MLST). All isolates showed resistance to nalidixic acid 40.5% (34/84), ciprofloxacin 11.9% (10/84), azithromycin 8.3% (7/84), and erythromycin 3.6% (3/84). Regarding the ten virulence genes detected, *cj1349*, *mviN* and *pldA* had the highest prevalence 100% (84/84), followed by *tlyA* 98.8% (83/84), *cadF* 97.6% (82/84), *ciaB* 71.4% (60/84), *hecA* and *hecB* 22.6% (19/84), *iroE* 15.5% (13/84) and *irgA* 10.7% (9/84), respectively. Three virulence genes were present among *A*. *butzleri* isolates of human diarrheal stool and food samples, with a significant difference observed among isolates; *hecB* [36% (9/25) and 8.6% (3/35)], *hecA* [36% (9/25) and 5.7% (2/35)], and *irgA* [24% (6/25) and 2.9% (1/35)] (*p* < 0.05), respectively. The *hecA* and *hecB* virulence genes functions are related to the mechanism of hemolysis, while *irgA* supports a bacterial nutritional requirement. MLST analysis of 26 *A*. *butzleri* isolates revealed that 16 novel STs exhibited high genetic diversity. The results of this study is useful for understanding potentially pathogenic and antimicrobial-resistant *A*. *butzleri* in Thailand. The pathogenic virulence markers *hecB*, *hecA*, and *irgA* have the potential to be developed for rapid diagnostic detection in human diarrheal stool. No significant relationships among STs and sources of origin were observed. Little is known about *A*. *butzleri*, the mechanism of action of these virulence genes, is a topic that needs further investigation.

## Introduction

Bacteria in the genus *Arcobacter* are emerging food-borne zoonotic pathogens. Recently, *Arcobacter butzleri* and *Arcobacter cryaerophilus* have been classified as microbial hazards to human health by the International Commission on Microbiological Specifications for Foods (ICMSF) [1–3]. *Arcobacter* spp. are slightly curved shape Gram-negative bacteria possessing one polar flagellum or bipolar flagella. The genus *Arcobacter* was first identified by Vandamme *et al*. [4] and currently includes 27 species [5]. Of the 27, *A*. *butzleri*, *A*. *skirrowii*, and *A*. *cryaerophilus* were reported to be associated with human foodborne diseases and isolated from human clinical stool specimens and blood cultures [1, 6–10]. Contaminated undercooked or raw meat i.e. chicken, pork, beef, shellfish, and water have been identified as major sources of infection [11–16]. Recently, a study in Thailand reported that 13% (9/70) of meals served in some restaurants in Bangkok were contaminated with *A*. *butzleri* [17]. Furthermore, *A*. *butzleri* was detected in 74% (54/73) of raw meat and poultry samples at the local market in Kanchanaburi province located in the western region of Thailand [18].

Antimicrobial susceptibility tests of *Arcobacter* using Etest, agar dilution, and disc diffusion have been reported [17, 19–21]. Macrolides (erythromycin and azithromycin) or fluoroquinolones (ciprofloxacin) are the recommended drugs of choice for treatment of *Arcobacter* infections [17, 20, 22]. Tetracyclines and aminoglycosides are alternative treatments for this infection in veterinary and human medicine to overcome resistance [20, 21, 23]. However, few studies investigating antimicrobial susceptibility of *Arcobacter* strains have been performed in Thailand [17, 18].

The genomic analysis of *A*. *butzleri* American Type Culture Collection (ATCC) 49616 revealed ten putative virulence genes: *cadF*, *cj1349*, *ciaB*, *hecA*, *hecB*, *mviN*, *pldA*, *tlyA*, *irgA*, and *iroE* [24]. Presence of these ten putative virulence genes in *Arcobacter* spp. isolates from human and food were determined by the PCR-based method [25–27]. The functions of each proposed virulence gene have previously been described in various pathogens. Genes *cadF* and *cj1349* encode for fibronectin-binding proteins that promote the binding of bacteria to intestinal cells [28]. The invasive genes *ciaB* and *Campylobacter* invasive antigen B contributes to host cell invasion through a secretion system [29]. *HecA* is a member of the filamentous hemagglutinin family and was reported to be involved in the attachment, aggregation, and epidermal cell killing of *Erwinia chrysanthemi* [30]. *HecB* encodes a hemolysin activation protein [24]. *MviN* can produce an essential protein required for peptidoglycan biosynthesis in *Escherichia coli* [31]. The phospholipase gene *pldA* encoding the outer membrane phospholipase A is associated with lysis of erythrocytes [32]. The hemolysin gene *tlyA* is also present in *Mycobacterium tuberculosis* and *Serpulina hyodysenteriae* [33]. The *irgA* and *iroE* genes are part of the functional components for iron acquisition and therefore is required for establishing and maintaining infections [34]. Virulence genes harboring in *A*. *butzleri* are mainly *cadF*, *cj1349*, *ciaB*, *mviN*, *pldA*, and *tlyA* with 100% detection in clinical (n = 84), food (n = 218) and environmental (n = 45) samples [25–26, 35] whereas *hecA*, *hecB* and *irgA* genes were identified at 21% (16/78), 68% (53/78), and 35% (27/78) from human specimens, respectively [25]. The virulence mechanisms and pathogenicity of *Arcobacter* spp. have rarely been demonstrated and is poorly understood. In Thailand, no evidence of *Arcobacter* virulence genes has been reported in human diarrheal, non-diarrheal stool, and food samples.

The genotypic diversity of *Arcobacter* spp. is often discriminated by molecular typing methods. Pulsed-field Gel Electrophoresis (PFGE), Amplified Fragment Length Polymorphism (AFLP), Enterobacterial Repetitive Intergenic Consensus (ERIC)-PCR, Matrix-Assisted Laser Desorption/Ionization Time-Of-Flight (MALDI-TOF) Mass Spectrometry (MS) and Multilocus Sequence Typing (MLST) methods have been used for *Arcobacter* typing from different strains isolated from different sources [36–40]. Miller *et al*. [39] proposed a MLST scheme for *Arcobacter* typing using seven housekeeping loci (*aspA*, *atpA*, *glnA*, *gltA*, *glyA*, *pgm*, and *tkt*). A total of 366 human-related *Arcobacter* isolates, from four continents and various sources were typed by MLST and found no association among STs and sources of origin or locations [25]. At present, this method currently has been identified as a valuable technique for genotyping and assessing the diversity of *Arcobacter* spp. in humans [36, 39]. This study aims to determine antimicrobial susceptibility patterns and virulence genes profiles of archived *Arcobacter* isolated from human stool and food samples. Subsequently, the genetic diversity of selected *Arcobacter* isolates was analyzed using MLST.

## Materials and methods

### Bacterial isolates

A total of 84 *A*. *butzleri* isolates from the Department of Bacterial and Parasitic Diseases, AFRIMS, Bangkok, Thailand were used in this study. *Arcobacter* spp. were previously isolated from human diarrheal (n = 25) and non-diarrheal (n = 24) stool samples, raw chicken (n = 15), raw beef (n = 11), raw pork (n = 8), and a chicken egg (n = 1) from 2001 to 2016 by the laboratory at AFRIMS. *Arcobacter* were identified by conventional phenotypic tests as described in Bodhidatta *et al*. [17]. All archived *Arcobacter* spp. isolates were grown on a blood agar plate (BAP; 5% sheep blood in *Brucella* agar, Becton, Dickinson and Company, Sparks, MD, USA) and incubated at 37°C in microaerobic condition for 24–48 h.

### Antimicrobial susceptibility testing

Antimicrobial susceptibility of *Arcobacter* isolates were performed by the Kirby-Bauer disc diffusion method [17, 23, 41]. *Staphylococcus aureus* ATCC 25923, *E*. *coli* ATCC 25922 were used as the reference strains. Eight antimicrobial discs (Becton, Dickinson and Company, Sparks, MD, USA) used in this study were azithromycin (AZM; 15 μg), ciprofloxacin (CIP; 5 μg), erythromycin (ERY; 15 μg), gentamicin (GM; 10 μg), kanamycin (KAN; 30 μg), nalidixic acid (NA; 30 μg), streptomycin (STR; 10 μg), and tetracycline (TE; 30 μg). The zone diameter of each *Arcobacter* isolate was interpreted by comparing with the zone diameter interpretive standards for *Enterobacteriaceae* and *S*. *aureus* according to the Clinical Laboratory Standards Institute (CLSI) [41]. Multidrug resistance was defined as acquired resistance to at least one antimicrobial agent in three or more antimicrobial drug classes [42].

### Detection of ten putative virulence genes of *Arcobacter* spp. by single PCR assay

Primers for *cadF*, *cj1349*, *ciaB*, *hecA*, *hecB*, *mviN*, *pldA*, *tlyA*, *irgA* and *iroE* genes amplification were obtained from previous studies [25, 26]. *A*. *butzleri* ATCC 49616 was used as a positive control. Briefly, the PCR mixture was prepared in a final volume of 25 μl per reaction on a PCR Thermocycler (Veriti 96 well Thermal Cycler, Applied Biosystems, Austin, TX, USA). The reaction mixture consisted of 1X PCR buffer, 0.2 mM dNTP, 50 μM specific primer set, 1.25 U Ampli-*Taq* Gold polymerase (Applied Biosystems, Austin, TX, USA), and 1 μl genomic DNA. The PCR parameters included initial denaturation at 94°C for 3 min, 32 cycles of 94°C for 45 s, 53°C for *ciaB*, *cj1349*, *mviN*, *pldA*, and *tlyA* genes; 55°C for *cadF*, *hecB*, and *iroE* genes; 56°C for *hecA*, and *irgA* genes for 45 s, 72°C for 45 s, and a final extension of 72°C for 3 min. Electrophoresis of PCR products in 1.0% agarose gel was performed and stained with ethidium bromide to visualize PCR fragment by using a transilluminator (Alpha Innotech, San Leandro, CA, USA).

### Multilocus sequence typing (MLST)

MLST scheme and primer sets of seven housekeeping gene loci are available at the *Arcobacter* MLST website (http://pubmlst.org/arcobacter). *A*. *butzleri* ATCC 49616 were used as positive controls of the PCR assays. The PCR mixture was prepared with 50-μls per reaction on a PCR Thermocycler (Veriti 96 well Thermal Cycler, Applied Biosystems, Austin, TX, USA). The reaction mixture consisted of 1X PCR buffer, 0.2 mM dNTP, 50 μM each primer set, 1 U *Taq* DNA polymerase (Qiagen Inc., Germantown, MD, USA), and 2 μl genomic DNA. The optimal PCR conditions were initial denaturation at 95°C for 2 min, then 35 cycles of 95°C for 45 s, 55°C for *pgm*; 57°C for *aspA*, *atpA*, *glnA*, *gltA*, and *tkt*; 59°C for *glyA* locus for 45 s, and 72°C for 30 s, and a final extension of 72°C for 10 min. Gel electrophoresis and visualization were performed as described above. The amplicons were purified by using Wizard^®^ SV Gel and PCR clean-up system (Promega, Madison, WI, USA) and were sequenced (1^st^ BASE, The Gemini, Singapore Science Park II, Singapore). Sequences were submitted to the Bacterial Isolate Genome Sequence Database (BIGSDB) [43] at the *Arcobacter* MLST website (http://pubmlst.org/arcobacter/).

### Statistical analysis

Association of virulence genes and antimicrobial susceptibility of *Arcobacter* spp. was analyzed by the Chi-Square test in the IBM SPSS Statistics 24 program (IBM, New York, NY, USA). The nucleotide sequences for MLST were aligned and checked for quality by using the Sequencher software version 5.4 (Gene Codes Corporation, Ann Arbor, MI, USA). The phylogenetic analysis and the Minimum Spanning Tree (MST) of *Arcobacter* isolates in Thailand were studied by using goeBURST implemented in PHYLOViZ [44] online at https://online.phyloviz.net.

## Results

### Antimicrobial susceptibility testing

A total of 84 *A*. *butzleri* isolates originating from human diarrheal (n = 25) and non-diarrheal stool (n = 24), and food samples (n = 35) were tested. The majority of isolates were resistant to NA at 40.5% (34/84) followed by CIP at 11.9% (10/84), AZM at 8.3% (7/84) and ERY at 3.6% (3/84). The resistance rate of *A*. *butzleri* isolates from human diarrheal and non-diarrheal stool, and food samples, the majority of the resistance was also to NA at 52% (13/25), 54.2% (13/24) and 22.9% (8/35), respectively. No resistance to aminoglycosides i.g. GM, KAN, and STR, and TE were detected (Table 1). No multidrug resistance was determined in all *Arcobacter* isolates.

**Table 1:** Resistance to antimicrobial agents of *Arcobacter butzleri* isolates from human diarrheal and non-diarrheal stool and food samples.

Antimicrobial agents	Disc content (μg)	No. (%) of isolates resistant to antimicrobial agents			
		Human diarrheal (n = 25)	Human non-diarrheal (n = 24)	Food (n = 35)	Total (N = 84)
**Macrolide**					
**Azithromycin**	15	3 (12)	4 (16.7)	0	7 (8.3)
**Erythromycin**	15	2 (8)	1 (4.2)	0	3 (3.6)
**Quinolone**					
**Ciprofloxacin**	5	2 (8)	4 (16.7)	4 (11.4)	10 (11.9)
**Nalidixic Acid**	30	13 (52)^a^	13 (54.2)^a^	8 (22.9)^a^	34 (40.5)
**Aminoglycoside**					
**Gentamicin**	10	0	0	0	0
**Kanamycin**	30	0	0	0	0
**Streptomycin**	10	0	0	0	0
**Tetracyclines**					
**Tetracycline**	30	0	0	0	0

^*a*^ Significantly different (Chi-square test; *p* < 0.05)

The percent resistant to NA in *A*. *butzleri* isolates in stool samples from human diarrheal (52%, 13/25) and non-diarrheal (54.2%, 13/24), were significantly higher than those isolates from food samples [(22.9%, 8/35), (*p* < 0.05)].

### Detection of ten putative virulence genes of *Arcobacter butzleri*

Among 84 *A*. *butzleri* isolates, the predominant virulence genes were *cj1349*, *mviN*, and *pldA* detected at 100% (84/84), followed by *tlyA* at 98.8% (83/84), *cadF* at 97.6% (82/84), *ciaB* at 71.4% (60/84), *hecA* and *hecB* at 22.6% (19/84), *iroE* at 15.5% (13/84), and *irgA* at 10.7% (9/84), respectively (Table 2).

**Table 2:** The prevalence of the ten putative virulence genes in *Arcobacter butzleri* isolates from various sources.

Source	n	No. (%) of isolates generating specific gene amplicon									
		Adhesins		O-Antigen	Invasins		Pore-forming toxins/ haemolysin			Iron uptake systems	
		*cadF*	*cj1349*	*mviN*	*ciaB*	*pldA*	*hecA*	*hecB*	*tlyA*	*irgA*	*iroE*
**Human diarrheal stool**	25	25 (100)	25 (100)	25 (100)	20 (80)	25 (100)	9^a^ (36)	9^a^ (36)	24 (96)	6^a^ (24)	6 (24)
**Human non-diarrheal stool**	24	24 (100)	24 (100)	24 (100)	16 (66.7)	24 (100)	8^a^ (33.3)	7 (29.2)	24 (100)	2 (8.3)	4 (16.7)
**Food**	35	33 (94.3)	35 (100)	35 (100)	24 (68.6)	35 (100)	2^a^ (5.7)	3^a^ (8.6)	35 (100)	1^a^ (2.9)	3 (8.6)
**Chicken eggs**	1	1 (100)	1 (100)	1 (100)	1 (100)	1 (100)	0	0	1 (100)	0	0
**Fresh beef**	11	10 (90.9)	11 (100)	11 (100)	5 (45.5)	11 (100)	1 (9.1)	1 (9.1)	11 (100)	1 (9.1)	1 (9.1)
**Fresh chicken meat**	15	15 (100)	15 (100)	15 (100)	10 (66.7)	15 (100)	0	0	15 (100)	0	0
**Fresh pork**	8	7 (87.5)	8 (100)	8 (100)	8 (100)	8 (100)	1 (12.5)	2 (25)	8 (100)	0	2 (25)
**Total**	84	82 (97.6)	84 (100)	84 (100)	60 (71.4)	84 (100)	19 (22.6)	19 (22.6)	83 (98.8)	9 (10.7)	13 (15.5)

^*a*^ Significantly different (Chi-square test; *p* < 0.05)

The prevalence of *hecA*, *hecB*, and *irgA* in *A*. *butzleri* isolates from human diarrheal stool samples [*hecA* 36% (9/25), *hecB* 36% (9/25), and *irgA* 24% (6/25), respectively] were significantly higher than those isolates from food samples [5.7% (2/35), 8.6% (3/35), and 2.9% (1/35), respectively] (*p* < 0.05). Furthermore, *hecA* in *A*. *butzleri* isolates from human non-diarrheal stool samples (33.3%, 8/24) was significantly higher than those isolates from food samples [(5.7%, 2/35) (*p* < 0.05)].

Among 84 isolates of *A*. *butzleri*, the most common virulence genes profiled was *cadF-cj1349-ciaB-mviN-pldA-tlyA*, which were detected in 48.6% (17/35) from food samples, 41.7% (10/24) from human non-diarrheal stools, and 28% (7/25) from human diarrheal stools. The common virulence genes detected in all *A*. *butzleri* were *cj1349*, *mviN*, and *pldA*. Only 14.3% (5/35) of *A*. *butzleri* isolates from food samples possessed at least 7 virulence genes whereas 56% (14/25) of *A*. *butzleri* isolates from human diarrheal stools possessed those genes. Only one *A*. *butzleri* isolate from raw beef harbored all ten virulence genes. Regardless of the sources, all *A*. *butzleri* isolates possessed potential virulence genes that can cause diarrheal diseases in humans (Table 3).

**Table 3:** The profiles of putative virulence genes of *Arcobacter butzleri* isolates from human diarrheal stool and non-diarrheal stool and food samples.

Profile of virulence genes	No. (%) of isolates		
	Human diarrheal stool (n = 25)	Human non-diarrheal stool (n = 24)	Food (n = 35)
Quintuple	4 (16)	2 (8.3)	13 (37.1)
*cadF-cj1349-mviN-pldA-tlyA*	3 (12)	2 (8.3)	11 (31.4)
*cadF-cj1349-ciaB-mviN-pldA*	1 (4)	0	0
*ciaB-cj1349-mviN-pldA-tlyA*	0	0	2 (5.7)
Sextuple	7 (28)	12 (50.1)	17 (48.6)
*cadF-cj1349-hecA-mviN-pldA-tlyA*	0	1 (4.2)	0
*cadF-cj1349-hecB-mviN-pldA-tlyA*	0	1 (4.2)	0
*cadF-cj1349-ciaB-mviN-pldA-tlyA*	7 (28)	10 (41.7)	17 (48.6)
**At least septuple**	**14 (56)**	**10 (41.8)**	**5 (14.3)**
Septuple	4 (16)	6 (25.1)	3 (8.5)
*cadF-cj1349-ciaB-hecB-mviN-pldA-tlyA*	2 (8)	1 (4.2)	1 (2.9)
*cadF-cj1349-ciaB-hecA-mviN-pldA-tlyA*	0	1 (4.2)	0
*cadF-cj1349-hecA-hecB-mviN-pldA-tlyA*	2 (8)	4 (16.7)	0
*cadF-cj1349-ciaB-mviN-pldA-tlyA-iroE*	0	0	2 (5.7)
Octuple	6 (24)	3 (12.5)	1 (2.9)
*cadF-cj1349-ciaB-hecA-hecB-mviN-pldA-tlyA*	3 (12)	0	1 (2.9)
*cadF-cj1349-ciaB-hecA-mviN-pldA-tlyA-iroE*	0	1 (4.2)	0
*cadF-cj1349-ciaB-irgA-mviN-pldA-tlyA-iroE*	3 (12)	2 (8.3)	0
Nonuple	4 (16)	1 (4.2)	0 (0)
*cadF-cj1349-ciaB-hecA-mviN-pldA-tlyA-iroE*-*irgA*	2 (8)	0	0
*cadF-cj1349-ciaB-hecA-hecB-mviN-pldA-tlyA-irgA*	1 (4)	0	0
*cadF-cj1349-ciaB-hecA-hecB-mviN-pldA-tlyA-iroE*	1 (4)	1 (4.2)	0
Decuple	0 (0)	0 (0)	1 (2.9)
*cadF-cj1349-ciaB-hecA-hecB-mviN-pldA-tlyA*- *irgA-iroE*	0	0	1 (2.9)

### Multilocus sequence typing (MLST) for *Arcobacter* species

Nucleotide sequences of seven housekeeping genes (*aspA*, *atpA*, *glnA*, *gltA*, *glyA*, *pgm*, and *tkt*) of 26 *Arcobacter* isolates were analyzed (Table 4). A total of 26 representative isolates of *Arcobacter* spp. were selected based on the presence of virulence genes, antimicrobial susceptibility patterns, sources of sample, location, and year of isolation for the MLST assay. Among the 26 *Arcobacter* isolates, 140 alleles and 23 STs were identified across all seven loci. A total of 32 new allele numbers and 16 new STs (ST576, ST582, ST583, ST585, ST591, ST592, ST612-ST621) were identified in the present study. The predominant new alleles was *pgm* (45.5%; 10/22), followed by *glyA* (34.8%; 8/23), *tkt* (27.8%; 5/18), *aspA* (18.2%; 4/22), *atpA* (15.8%; 3/19), *glnA* (10.5%; 2/19), and *gltA* (5.9%; 1/17).

**Table 4:** Multilocus sequence typing results of the 26 *Arcobacter butzleri* isolated from human diarrheal and non-diarrheal stool and food samples.

No.	Code of isolate	ST	Allele ID of housekeeping genes							Source of sample	Location	Year of isolation
			*aspA*	*atpA*	*glnA*	*gltA*	*glyA*	*pgm*	*tkt*			
**1**	AF-ARCO-FC-77	30	5	12	7	9	33	7	24	Food (raw chicken)	Bangkok	2003
**2**	AF-ARCO-FC-78	31	5	12	11	26	36	30	24	Food (raw chicken)	Bangkok	2003
**3**	AF-ARCO-FC-79	94	21	22	21	24	48	27	25	Food (raw chicken)	Bangkok	2003
**4**	AF-ARCO-FC-75	74	19	17	17	20	26	22	19	Food (raw chicken)	Bangkok	2003
**5**	AF-ARCO-FP-80	3	2	2	24	27	112	35	20	Food (raw pork)	Bangkok	2003
**6**	AF-ARCO-HD-17	130	34	12	2	34	58	46	38	Human diarrheal stool	Trang	2006
**7**	AF-ARCO-HD-19	**582**	38	35	26	20	165	51	4	Human diarrheal stool	Trang	2006
**8**	AF-ARCO-HD-88	130	34	12	2	34	58	46	38	Human diarrheal stool	Trang	2006
**9**	AF-ARCO-HD-56	**612**	30	5	5	30	**615**	50	40	Human diarrheal stool	Chiang Rai	2008
**10**	AF-ARCO-HD-57	**612**	30	5	5	30	**615**	50	40	Human diarrheal stool	Chiang Rai	2008
**11**	AF-ARCO-HD-74	**615**	40	17	2	63	54	**325**	31	Human diarrheal stool	Bangkok	2008
**12**	AF-ARCO-ND-58	**616**	3	3	30	15	**598**	**330**	4	Human non-diarrheal stool	Chiang Rai	2008
**13**	AF-ARCO-ND-59	**585**	173	41	19	6	**599**	77	**263**	Human non-diarrheal stool	Chiang Rai	2008
**14**	AF-ARCO-ND-60	**617**	80	67	49	30	524	263	**267**	Human non-diarrheal stool	Chiang Rai	2008
**15**	AF-ARCO-HD-63	**613**	**285**	66	1	20	**601**	**326**	**261**	Human diarrheal stool	Nakhon Ratchasima	2009
**16**	AF-ARCO-ND-65	**583**	25	**194**	127	32	52	42	20	Human diarrheal stool	Pisanulok	2009
**17**	AF-ARCO-HD-70	166	50	40	19	45	165	68	48	Human diarrheal stool	Surajthani	2009
**18**	AF-ARCO-ND-64	**618**	**292**	32	152	34	46	**327**	36	Human non-diarrheal stool	Surajthani	2009
**19**	AF-ARCO-ND-66	130	34	12	2	34	58	46	38	Human non-diarrheal stool	Surajthani	2009
**20**	AF-ARCO-ND-67	**592**	39	**196**	**180**	37	73	**322**	**264**	Human non-diarrheal stool	Surajthani	2009
**21**	AF-ARCO-ND-68	**619**	**293**	42	3	20	**596**	**331**	4	Human non-diarrheal stool	Pisanulok	2009
**22**	AF-ARCO-ND-69	**620**	42	25	7	44	**616**	**332**	2	Human non-diarrheal stool	Surajthani	2009
**23**	AF-ARCO-ND-71	**576**	30	5	9	30	120	35	4	Human non-diarrheal stoool	Surajthani	2010
**24**	AF-ARCO-ND-72	**621**	3	31	1	20	545	**326**	**261**	Human non-diarrheal stool	Bangkok	2013
**25**	AF-ARCO-HD-73	**614**	81	62	26	144	**597**	**328**	**260**	Human diarrheal stool	Bangkok	2014
**26**	AF-ARCO-HD-90	**591**	**291**	**195**	**173**	**198**	**602**	**318**	222	Human diarrheal stool	Chonburi	2016

Boldface entries represent new alleles and STs

The minimal spanning tree (MST) of seven housekeeping genes loci (3,341 bp) was constructed online at https://online.phyloviz.net to find a relationship among the 26 studied isolates, using the 120 isolate database (retrieved Jun 19, 2017, from http://pubmlst.org/arcobacter/), five ATCC and two reference strains. The reference strains consisted of *A*. *butzleri* ATCC 49616, *A*. *skirrowii* ATCC 51400, *A*. *cryaerophilus* ATCC 49615, *Arcobacter cibarius* LMG 21996, *Arcobacter thereius* LMG 24486, *Campylobacter jejuni* ATCC 700819, and *Helicobacter pylori* ATCC 26695. Overall, all STs of *A*. *butzleri* were clustered in one group, whereas the other species were split up and linked between ST111 of *A*. *butzleri* and *A*. *skirrowii* ATCC 51400. Among 26 *Arcobacter* isolates, the ST-94, ST-130 and ST-166 (Table 4) are dected in raw chicken and human diarrheal stool which related to the mixed sources of samples including human diarrheal stool and non-diarrheal stool and food samples obtaining from the *Arcobacter* database (http://pubmlst.org/arcobacter/). In the present study, ST, sources of origins, location, or year were not related, however few isolates from the human diarrheal stool and food samples (chicken offal or meat and pork offal or meat) shared identical ST(s).

**Fig 1:**
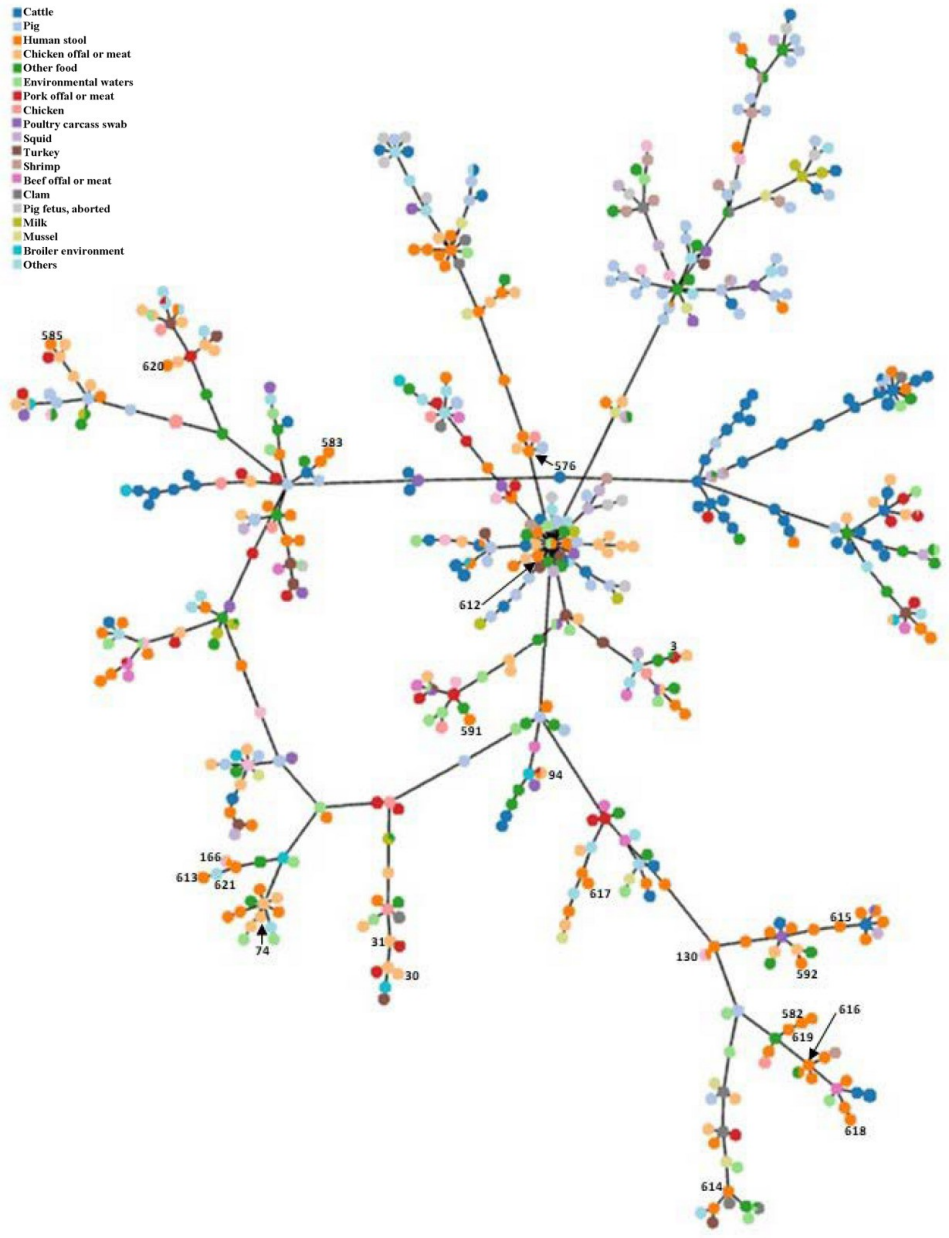
The Minimum Spanning Tree (MST) of all *Arcobacter* isolates in pubmlst database. This tree was constructed based on the concatenated sequences of seven housekeeping genes loci (3,341 bp) of 867 *Arcobacter* isolates obtained from the present study (n = 26) and the database (n = 841). The number beside the node indicates ST in the present study.

In accordance with the MST, 26 *Arcobacter* isolates from this study and entire 867 *Arcobacter* isolates obtained from the *Arcobacter* database (retrieved Jun 19, 2017, from http://pubmlst.org/arcobacter/) (Fig 1) was constructed online at https://online.phyloviz.net. A total of 20 source categories were found in the worldwide *Arcobacter* MLST database. Taken together, only species-related including *A*. *butzleri*, *A*. *cryaerophilus*, *A*. *skirrowii*, *A*. *cibarius*, and *A*. *thereius* obtained from the database formed the clusters. No association of sources and genetic profiles of isolates were observed for this organism.

## Discussion

In a previous study, the prevalence of *Arcobacter* spp. showed that the overall percentage of resistance to ciprofloxacin ranged from 5.7–14.8% and 5.6–19.2% for erythromycin [45]. In this study, we found a lower resistance rate to ciprofloxacin at 11.9% (10/84) and erythromycin at 3.6% (3/84) in *A*. *butzleri* isolates. The resistance rate to ciprofloxacin was 8.0% (2/25) of the *A*. *butzleri* isolated from human diarrheal stool samples and 11.4% (4/35) of the *A*. *butzleri* isolated from food samples, which was consistent with previous reports in Thailand [17]. *A*. *butzleri* isolates from human diarrheal stool samples showed lower resistance to ciprofloxacin at 3.3% (2/61) in Belgium [22] and 7.4% (2/27) in Spain [46], whereas resistance to ciprofloxacin was significantly higher in the USA than those in Asia (*P* < 0.001) [45]. Results from our study showed that *A*. *butzleri* isolates from human diarrheal stool samples had low resistance to erythromycin at 8.0% (2/25) and none of the *A*. *butzleri* isolates from food samples were resistant to erythromycin. However, previous studies showed that isolates from the human diarrheal stool and food samples were erythromycin-resistant at 4–21% and 0–12%, respectively [19, 22, 46–50]. *A*. *butzleri* showed 100% (84/84) susceptibility to gentamicin, kanamycin, streptomycin, and tetracycline in the present study. These results are similar to the previous reports which suggested that the aminoglycosides (gentamicin, kanamycin, and streptomycin) and tetracyclines (tetracycline) can be used as alternative drugs of choice [22, 26, 47, 49]. A previous study showed 93.8% (75/80) multidrug resistance in *A*. *butzleri* isolates [50] and 68.9% (440/638) in *Arcobacter* spp. [45]. Nevertheless, no multidrug resistance was observed in our study. Antimicrobial susceptibility in this study indicated that fluoroquinolones and macrolides are currently suitable treatments for *Arcobacter* infections in Thailand.

This study is the first to report the detection of ten virulence genes, including *cadF*, *cj1349*, *ciaB*, *hecA*, *hecB*, *mviN*, *pldA*, *tlyA*, *irgA*, and *iroE* of *Arcobacter* spp. isolates in Thailand. The presence of *A*. *butzleri* virulence genes showed similar results to Karadas *et al*. [26] who studied ten virulence genes to investigate the potential pathogenic *A*. *butzleri* isolated from food samples, and water. Almost all *A*. *butzleri* isolates possessed genes *cadF*, *cj1349*, *ciaB*, *mviN*, *pldA*, and *tlyA*, and rarely possessed *hecA*, *hecB*, *irgA*, and *iroE* [21, 27, 35, 46, 51–53]. In particular, genes *hecA* 36% (9/25), *hecB* 36% (9/25), and *irgA* 24% (6/25) were detected in human isolates at a higher rate than those from food samples [5.7% (2/35), 8.6% (3/35), and 2.9% (1/35), respectively] (*p* < 0.05). This result is consistent with previous studies [25, 35] implying that these genes might play an important role associated with the human host.

Genetic diversity of *Arcobacter* spp. was determined by MLST. The MLST of 26 *A*. *butzleri* isolates, 140 alleles, and 23 STs were identified, with 16 novel STs and 22.9% (32/140) new alleles being reported. Miller *et al*. [39] reported no association between STs from clinical, food, and environmental samples with a host or geographical source. Additionally, MLST revealed the genetic diversity of *A*. *butzleri* isolates from various samples and showed no association of alleles and STs with animal fecal samples [54], products of animal origin [55], food and contact surfaces [40]. Moreover, ST-617 from our study in Thailand was clustered with samples from the University Hospital Sant Joan de Reus (n = 3), the University Hospital Joan XXIII (n = 4) in Spain, and one STs from the USA [46]. Furthermore, *A*. *butzleri* isolates with ST-94 and ST-166 were found in both human diarrheal stool samples and chicken offal or meat samples in Thailand. The highest of STs in this study was ST-130, the result was similar to the high STs in Thailand (there were four isolates in each ST-56, ST-94, ST-117, and ST-130). The ST-130 was previously identified in *A*. *butzleri* isolated from human diarrheal stool sample in Vietnam (2002) and human non-diarrheal stool sample in Thailand (2002) [39] whereas three isolates of ST-130 in our study were isolated from two human diarrheal stool samples in Trang province (2006) and one human non-diarrheal stool sample at Surajthani province (2009), Thailand. Our MLST, ST-94 and ST-166 presented that raw chicken is a possible source of *Arcobacter* transmission (http://pubmlst.org/arcobacter/). However, no distinct correlation was observed between the origin of the sample and the geographical location. Also, the results from previous studies showed the persistence of the same ST from the same source, indicating possible cross-contamination between food and environmental sites [56–58]. In the present study, only 26 isolates were analyzed, the range of allelic density (number of alleles/number of strains) was 65.4% (17/26) at the *gltA* locus and 88.5% (23/26) at the *glyA* locus, whereas worldwide allelic density for *glnA* locus is 17.9% (155/867) and 48.4% (420/867) for *glyA* locus. The highest allelic density of *A*. *butzleri* was observed at 88.5% (23/26) for *glyA* and followed by 84.6% (22/26) for *pgm*. These findings coincided with the MLST study that the highest allelic density of *A*. *butzleri* was 68% (21/31) for *glyA* and followed by 54% (13/24) for *pgm* in the Northern part of Spain [55], and 28.2% (11/39) for *glyA* and 25.6% (10/39) for *pgm* in the United Kingdom [54]. This report of the high allelic density at the *glyA* and *pgm* loci is consistent with the first MLST study for *A*. *butzleri* [39]. Furthermore, the allelic density of 26 *A*. *butzleri* isolates in Thailand showed high diversity that ranged from 65.4% (17/26) of *gltA* to 88.5% (23/26) of *glyA*.

Antimicrobial resistant strains of *A*. *butzleri* in meats should be monitored for contamination and for antimicrobial resistance strains in food products. These pathogenic virulence markers such as *hecB*, *hecA*, and *irgA* have the potential to be developed for rapid diagnostic detection in human diarrheal stool. The *glyA* and *pgm* loci are important for studying the genetic diversity of *Arcobacter* spp. The collection and analysis of a larger sample size of *A*. *butzleri* isolates will generate a more comprehensive epidemiological understanding of this microorganism that is emerging as an important foodborne illness.
